# Supplementary material for: End Sequence Analysis Toolkit (ESAT) expands the extractable information from single-cell RNA-seq data
Source: Genome Res. 2016 Oct;26(10):1397–410. doi: 10.1101/gr.207902.116 (PMC5052061; doi:10.1101/gr.207902.116)
Supplement: Supplemental Material [file supp_gr.207902.116_Supp_Materials.tar.gz › ESAT-esat_v0.1/doc/html/functions_q.html]

ESAT: Class Members


ESAT


- Main Page
- Related Pages
- Packages
- Classes
- Files

- Class List
- Class Index
- Class Hierarchy
- Class Members

- All
- Functions
- Variables
- Enumerator

- a
- b
- c
- d
- e
- f
- g
- h
- i
- j
- k
- l
- m
- n
- o
- p
- q
- r
- s
- t
- u
- v
- w
- z


All Classes Namespaces Files Functions Variables Enumerator Pages

Here is a list of all class members with links to the classes they belong to:

### - q -

- QCAgilentResults()
  : broad.pda.geneexpression.agilent.QCAgilentResults
- qr()
  : Jama.Matrix
- QRDecomposition()
  : Jama.QRDecomposition
- quantile()
  : broad.core.math.Statistics
- quantileNormalize()
  : broad.core.datastructures.MatrixWithHeaders
- quantileNormalizeColumns()
  : broad.core.datastructures.MatrixWithHeaders
  , Jama.Matrix
- query()
  : broad.core.datastructures.JCSCache
  , broad.pda.samtools.SAMPairedEndFileReader
- QUERY
  : broad.pda.seq.rap.DistanceToBED
- query()
  : umms.core.readers.PairedEndReader
- queryStarts
  : umms.core.annotation.PSLRecord
- QUEUE
  : broad.pda.seq.rap.BuildRatioNullDistribution
  , broad.pda.seq.rap.CollectAnnotationEnrichments


---

Generated on Fri Aug 15 2014 15:11:16 for ESAT by  

 1.8.7
